# Interactions of Destruxin A with Silkworms’ Arginine tRNA Synthetase and Lamin-C Proteins

**DOI:** 10.3390/toxins12020137

**Published:** 2020-02-22

**Authors:** Jingjing Wang, Qunfang Weng, Fei Yin, Qiongbo Hu

**Affiliations:** Key Laboratory of Bio-Pesticide Innovation and Application of Guangdong Province; College of Agriculture, South China Agricultural University, Guangzhou 510642, China

**Keywords:** Destruxins, *Bombyx mori*, BmArgRS, BmLamin-C, RNA helicase, binding protein

## Abstract

Destruxin A (DA), a cyclodepsipeptidic mycotoxin produced by entomopathogenic fungus *Metarhizium anisopliae*, has good insecticidal activity and potential to be a new pesticide. However, the mechanism of action is still obscure. Our previous experiments showed that DA was involved in regulation of transcription and protein synthesis and suggested that silkworms’ arginine tRNA synthetase (BmArgRS), Lamin-C Proteins (BmLamin-C) and ATP-dependent RNA helicase PRP1 (BmPRP1) were candidates of DA-binding proteins. In this study, we employed bio-layer interferometry (BLI), circular dichroism (CD), cellular thermal shift assay (CETSA), and other technologies to verify the interaction of DA with above three proteins in vitro and in vivo. The results of BLI indicated that BmArgRS and BmLamin-C were binding-protein of DA with K_D_ value 5.53 × 10^−5^ and 8.64 × 10^−5^ M, but not BmPRP1. These interactions were also verified by CD and CETSA tests. In addition, docking model and mutants assay in vitro showed that BmArgRS interacts with DA at the pocket including Lys228, His231, Asp434 and Gln437 in its enzyme active catalysis region, while BmLamin-C binds to DA at His524 and Lys528 in the tail domain. This study might provide new insight and evidence in illustrating molecular mechanism of DA in breaking insect.

## 1. Introduction

**Figure 1:**
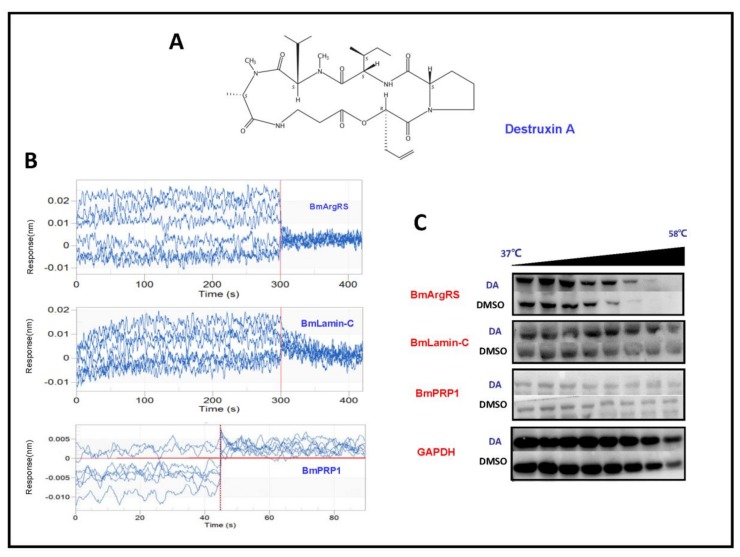
Profiles of interaction between Destruxin A (DA) with BmArgRS, BmLamin-C and BmPRP1 (**A**) The structure of Destruxin A. (**B**) BLI analysis showed the interactions of DA with BmArgRS and BmLamin-C but not BmPRP1 in vitro. (**C**) Cellular thermal shift assay (CETSA) results showed the interations of DA with BmArgRS and BmLamin-C in vivo.

Destruxin A (DA, Figure 1A) isolated from entomopathogenic fungus *Metarhizium anisopliae* is a cyclodepsipeptidic mycotoxin with insecticidal, antifeedant, and anti-immunity effects on host insects [1,2]. It is also reported that DA is a key pathogenic factor of *M. anisopliae* against insects [3]. DA is considered as a new potential pesticide to contract researchers interesting. For this, it is necessary to elucidate the targets of DA acting on insects. In the past decade, several studies indicated that DA changes the morphology of hemocytes and brings on equilibrium chaos of intra- and extra- cellular hydrogen and calcium ion in *Bombyx mori* [4,5], and regulates immune related gene expression [6,7]. Recently, a few DA binding-proteins in silkworm were found [8,9,10]. However, these results are not enough to explain the mechanisms of DA against insects.

To discover the DA target molecules, we conducted experiments based on drug affinity responsive target stability (DARTS) [11] in Bm12 cells; nuclear membranes protein Lamin-C (BmLamin-C), arginine tRNA synthetase (BmArgRS), and ATP-dependent RNA helicase PRP1 (BmPRP1) were detected. These three proteins have been studied thoroughly in human and model organisms to date. Summarily, Lamin-C belongs to nuclear intermediate filament proteins which provides mechanical stability, organizes chromatin and regulates transcription and other nuclear activities [12]. Moreover, recent studies have shown that Lamin-C plays role in development, tissue responsing to mechanical, reactive oxygen species, and thermal stresses [7]. ArgRS is significant in protein synthesis process, which provides arginine and maintaining fidelity in peptide chain extension [13,14]. While PRP1 has the functions of pre-mRNA-splicing and its fidelity of recognition [14,15]. However, these proteins have been rarely studied in *B. mori* and related biological pathways were never associated with DA target research before. In this study, we will validate the interaction of DA with these proteins in vivo and vitro. The results will provide new insights on better understanding for the DA-binding proteins.

## 2. Results

### 2.1. Interactions of Destruxin A with Three Proteins by BLI and CETSA Analysis

In order to evaluate whether BmLamin-C, BmArgRS and BmPRP1 proteins interact with DA, we conducted the experiments of bio-layer interferometry (BLI) in vitro and cellular thermal shift assay (CETSA) in vivo. In BLI assays, the results indicated that the responses of BmArgRS and BmLamin-C proteins were positively correlated with DA concentrations. There were affinity constant (K_D_) values of 5.53 × 10^−5^ and 8.64 × 10^−5^ M respectively for DA with BmArgRS and BmLamin-C (Table 1, Figure 1B). However, the results showed that BmLamin-C is not a DA-binding protein, because a significant correlation between DA concentration and response of BmPRP1 was not detected. Meanwhile, the results of CETSA experiments exhibited that DA induced the thermal stability shift of BmArgRS and BmLamin-C but not BmPRP1 (Figure 1C), which suggested that the two proteins interact with DA in vivo. Obviously, both the BLI and CETSA experiments provided in vitro and in vivo evidences for that DA binds to BmArgRS and BmLamin-C but not BmPRP1.

### 2.2. Key Sites of Interaction of DA with BmArgRS and BmLamin-C

**Figure 2:**
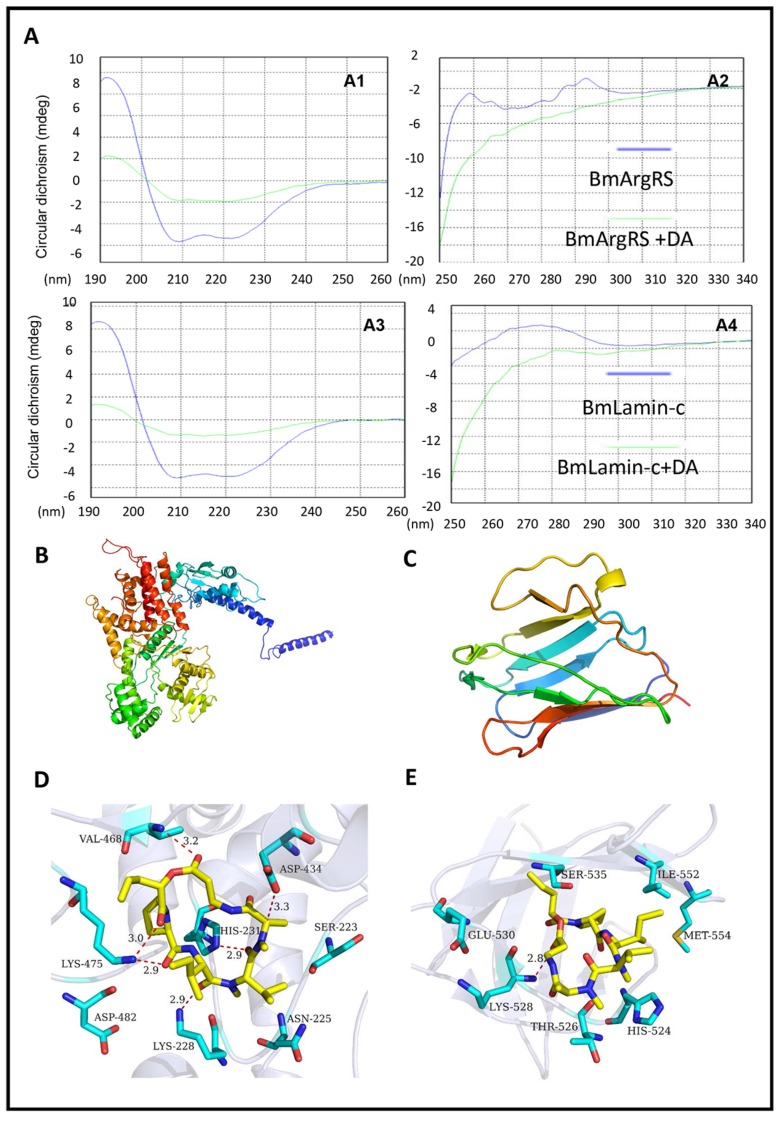
Structural evidences of interaction between DA with BmArgRS and BmLamin-C. (**A**) Profiles of circular dichroism tests indicating the effects of DA on proteins secondary structures. **A1**, **A2**, 190–260 nm and 250–340 nm ultraviolet region of BmArgRS interact with DA respectively. **A3**, **A4**, 190–260 nm and 250–340 nm ultraviolet region of BmLamin-C interact with DA respectively. (**B**,**C**) Homologous modeling of BmArgRS and BmLamin-C respectively. (**D**,**E**) Binding pose of DA with BmArgRS and BmLamin-C respectively.

In the circular dichroism (CD) tests, the scanning results in 190–260 nm ultraviolet region indicated the CD shifts of DA-treated BmArgRS and BmLamin-C, which suggest that DA damages the α-helixes of the two proteins (Figure 2A). Meanwhile, in the 250–340 nm ultraviolet scanning, the protein CD changes caused by DA were found, which indicated that DA brings on the transformation of disulfide bonds and some side chains of BmArgRS and BmLamin-C (Figure 2A). These data provide the evidences for the interactions of DA with BmArgRS and BmLamin-C.

Furthermore, in analysis of homologue modeling (Figure 2B,C), DA interacts with BmArgRS and BmLamin-C with the scores −9.71 and −8.08 kcal/mol, respectively. The molecular docking predicted that there are many hydrogen bonds between DA and BmArgRS, which provide a pocket consisted of Lys228, His231, Gln437, Lys475, Val468, and Asp434 to DA binding (Figure 2D). However, there is only a hydrogen bond between DA and Lys528 in BmLamin-C (Figure 2E), although a pocket formed by His524, Thr526, Lys528, Glu530, Ser535, Ile552, and Met554 was predicted.

**Figure 3:**
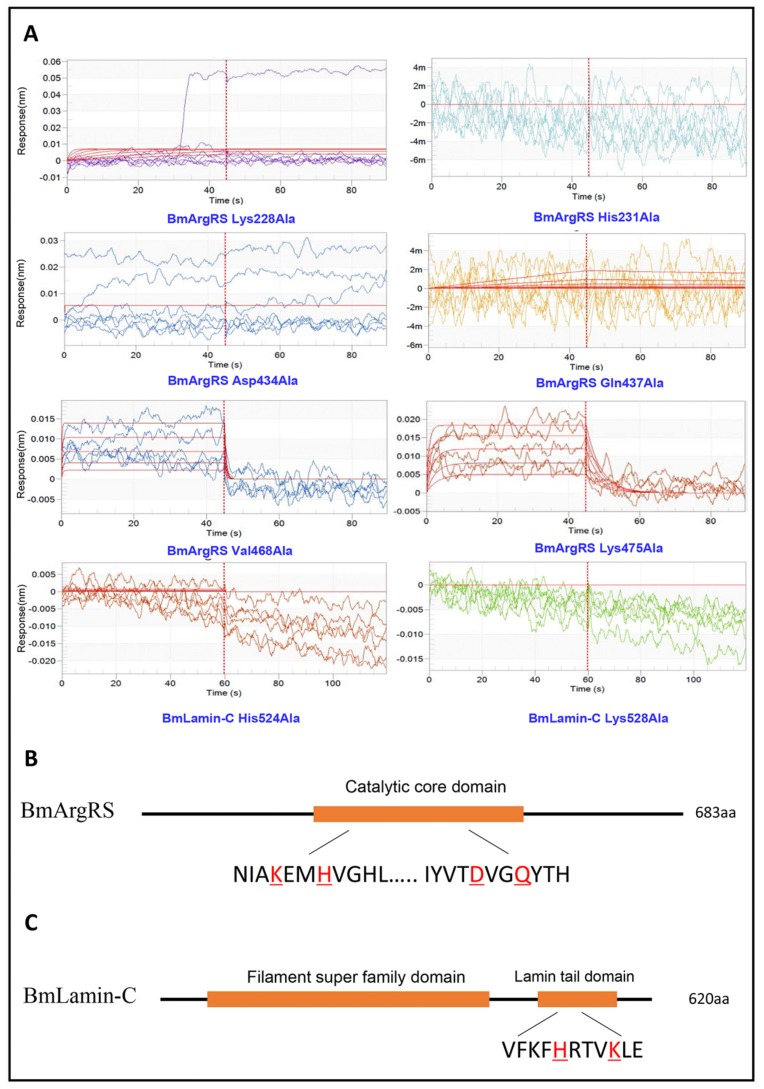
Key amino acid sites for interaction of DA with BmArgRS and BmLamin-C by BLI tests. (**A**) The interactions of DA with the mutants of BmArgRS and BmLamin-C. (**B**,**C**) Sketch of the domains of BmArgRS and BmLamin-C and key amino acid sites for DA binding.

Based on the analysis above, each of amino acids of comprising hydrogen bond was mutated to alanine and the interactions of all mutant proteins with DA were investigated through BLI assay. Interestingly, in BmArgRS, 4 mutants, His231Ala, Lys228Ala, Asp434Ala, and Gln437Ala, displayed no interaction with DA (Figure 3). However, the interactions were still found in the mutants Val468Ala and Lys475Ala with DA, because the K_D_ values of 5.3 × 10^−5^ and 5.71 × 10^−6^ were respectively recorded, which were at same level compared with wild type (Figure 3). Those illustrated that DA binds to BmArgRS in the pocket including Lys228, His231, Asp434, and Gln437. Strikingly, the interaction site is just located in the conserved enzyme active catalysis domain of BmArgRS. Likewise, the His524Ala and Lys528Ala mutants of BmLamin-C exhibited no interactions with DA, which implied that Lamin tail domain is the DA-binding site (Figure 3).

### 2.3. Gene Expression Levels of Three Proteins in Bm12 Cells

**Figure 4:**
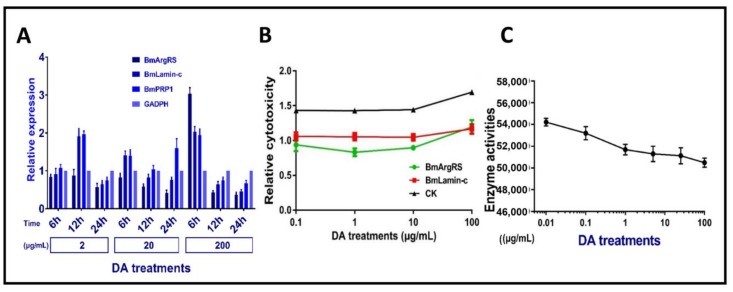
Effects of DA on BmArgRS and BmLamin-C of Bm12 cell. (**A**) Analysis of gene expression under DA stress by qPCR test. (**B**) Cytotoxicity of DA against Bm12 cells by RNAi treatments. (**C**) Enzyme activity of BmArgRS inhibited by DA.

We investigated DA dosage- and time-depend affecting expression three genes in Bm12 cells. The results indicated that there were no obvious relations between genes expression levels of three proteins in DA dosage- and time-depend manner. Totally, these genes had up-regulation of <2 folds, only BmArgRS was up-regulated by 3-fold in relative high dosage 200 μg/mL at 6 h post-treatment (Figure 4A). It is suggested that DA only leads to mild changes in gene expression levels of the three proteins.

### 2.4. Changes of DA Cytotoxicity and BmArgRS Enzyme Activity in Bm12 Cells

The results indicated that the toxicities of DA against Bm12 cells were decreased by more than 50% after the genes of *BmArgRS* and *BmLamin-C* were knocked down (Figure 4B). In addition, the enzyme activity of BmArgRS was decreased under DA exposure, because a negative correlation between enzyme activity and DA dosage was found (Figure 4C). This might explain that DA binds to enzyme active center of BmArgRS so as to inhibit its catalytic function.

## 3. Discussion

For research of small molecular drugs, it is of great importance to elucidate the target protein in special cells or tissues. In this study, we conducted the experiments of BLI, CETSA, qPCR, RNAi, and enzyme activity, as well as modeling and docking analysis. As results, we carefully validated that BmArgRS and BmLamin-C are the DA-binding proteins and elucidated their interaction modes. Obviously, it is significant for further developments of DA-like drugs and researches of target proteins. However, BmPRP1 was not DA-binding protein. In fact, similar to other RNA helicases, BmPRP1 was an important component of constitution of stress granules (SGs) under stimuli [16], while SGs, composed of mRNA and ribosomal subunit, expression initial factor and RNA-binding proteins, appear in cellular stress conditions such as hypoxia, oxidative stress, and virus infection so as to stall translation and expression for energy saving [17]. Interestingly, in our previous study, we found a DA-binding protein (BmTudor-sn) was also component of SGs [8]. In addition, there is a report showed that destruxin can induce oxidative stress in insect [18]. Therefore, it is more likely that DA injures cells in sorts of unknown mechanism to trigger mRNA translation and protein synthesis of related SGs components such as BmPRP1. It probably leads to a false-positivity of BmPRP1 in DARTS experiments.

Aminoacyl tRNA synthetases play an important role in protein synthesis and are usually considered as medicine targets [19,20,21]; among them, ArgRS is involved in the formation of arginyl-tRNA^Arg^ complex in peptide chain extension [22]. Sequence analysis indicates that BmArgRS contains a catalytic core domain of arginyl-tRNA synthetases in the region of 216–481. Interestingly, in this study, we proved that DA binds to a pocket of BmArgRS in the conserved active catalysis sites. It might be speculated that DA suppresses the synthesis of some proteins containing arginine, especially the arginine rich basic proteins (histone), subsequently causes and leads to cells chaos in insects. Our research results give some clues to development of ArgRS inhibitors. Although DA interacts with BmArgRS at lower level as 10^−5^ M, more other chemicals with higher bioactivity can be found on the basis of the quantitative structure-activity relationship (QSAR) of molecules and BmArgRS.

Lamins-C has many functions which usually acts as intermediate filament proteins providing stability and strength to cells, as supporting (scaffolding) components of the nuclear envelope regulating the movement of molecules into and out of the nucleus, and as a role in regulating the activity (expression) of certain genes [12]. Previously, researchers found out that DA showed no affinity to nucleus, but was abundant in cytosol [23]. As above indicated, nonpolar DA might access into nucleus at inner membrane to bind BmLamin-C but does not result in mechanical stress. In cytoplasm, lamins mainly function as response to eliminate heat shock and oxidative stress [7]. Interestingly, DA induced heat shock proteins upregulation and oxidative stress in cells. It is suggested that DA binds to BmLamin-C leading to maintain unsuitable environment in cells which constant to previous study that DA acts as immunosuppressor [2]. Intriguingly, C-terminal lamin tail domain is highly conserved and contains immunoglobulin fold. However, researches of Lamin-C were insufficient in *B. mori*, so it is hard to in-depth study in function with DA and make an assumption. But it provides new evidences and insight to further study owing to several nuclear related candidate proteins we have.

Reviewed in previous research on molecular mechanism of DA, the widely accepted hypothesis is that DA acts as immunosuppressor to inhibit innate immune system when *M. anisopliae* infected host. However, traditional molecular mechanism of DA target sites such as hemocyte and hemolymph are obscure. Here, we can speculate that DA stresses and leads to cell in uncomfortable environment and the meantime DA binds to those stress induced proteins to repress host defense as an immunosuppressor. Because we previously found that DA bond to heat shock protein [10], stress granule protein BmTudor-sn [8] and immunophilin peptidyl-prolylcis-transisomerase (BmPPI) [9], and interacted with BmArgRS to inhibit protein synthesis and bond to stress support protein BmLamin-C as well in this study.

In conclusion, we investigated the interaction between DA and three candidate proteins, and found that BmArgRS and BmLamin-C are DA-binding proteins but not BmPRP1. In addition, structurally, DA interacts with BmArgRS at Lys228, His231, Gln437 and Lys475 in catalysis active domain and with BmLamin-C at Lys528 and His524 in lamin tail domain. The above findings would offer new insight and evidence to DA target protein discovery.

## 4. Materials and Methods

### 4.1. Cell Culture and Destruxin A

The *Bombyx mori* Bm12 cell line was donated by our colleague Cao Yang (College of Animal Science, South China Agricultural University) and cultured in Grace’s culture medium (Hyclone, Pittsburgh, MA, USA) and 10% fetal bovine serum (Gibco, Waltham, MA, USA). Cells were cultured at 27 °C and maintained at over a period of 2–4 days.

Destruxin A (DA) was isolated and purified from the *Metarhizium anisopliae* var. *anisopliae* strain MaQ10 in our laboratory [24]. Stock solution is 10,000 µg/mL in dimethyl sulfoxide (DMSO, Sigma-Aldrich, Darmstadt, Germany).

### 4.2. Bio-Layer Interferometry (BLI)

All proteins were prepared by expression in *E. coli*, and were tagged with His-tag and purified by nickel affinity chromatography. Protein accession number of BmArgRS, BmLamin-C, and BmPRP1 are XP_004931696.1, XP_004930078.1, and XP_004926349.1, respectively.

BLI analysis was performed on a ForteBio OctetQK System (K2, Pall Fortebio Corp, Menlo Park, CA, USA) [25]. Generally, the protein samples were coupled with a biosensor for immobilization. Dilutions of DA were used for treatment. PBST buffer (0.05% Tween20, 5% DMSO) was used for the running and dilution buffers. The working procedure was baseline for 60 s, association for 60 s, and dissociation for 60 s. Finally, the raw data were processed with Data Analysis Software (9.0, Pall ForteBio Corp, Menlo Park, CA, USA).

### 4.3. Cellular Thermal Shift Assay (CETSA)

The Bm12 cell line was used to conduct the CETSA experiments [26]. Firstly, Bm12 cells were treated with 100 µg/mL DA and divided into 8–10 aliquots. After heated at 37–58 °C at 3 min, cells were lysed by a freeze-thawcycle. After centrifugated, the supernatants of the lysed cells were used for the western blot analysis.

### 4.4. RNAi and Toxicity Assessment RNAi

SiRNAs were prepared by synthesis in vitro. The sequence of BmLamin-C siRNA: 5′-GCUGAUACCCGUAAGACUUTT-3′ and 5′-AAGUCUUACGGGUAUCAGCTT-3′. The sequence of BmArgRS: 5′-GCGAUCAAGAAGGAAGCUATT-3′ and 5′-UAGCUUCCUUCUUGAUCGCTT-3′. SiRNA and FuGENE (Promega, Beijing, China) transfection reagent were each diluted in serum-free medium and then mixed. The mixture was added to Bm12 cells and DA treatments. Toxicity assessment was performed by LDH-Glo™ Cytotoxicity Assay (Promega, Beijing, China) and detected in Synergy™ H1 (BioTek, Winooski, VT, USA).

### 4.5. Survey of Gene Expression

Expression of target gene was measured by RT-qPCR. Primer of BmLamin-C: 5′-CTTCACCACGGCTCTGCTCAAC-3′ and 5′- TGCGGCAATTCTCTTCACCTTCG-3′. Primer of BmArgRS: 5′-GAGGTTAGAAGAGCGAACCACCAG-3′ and 5′-CGCCGCTGAAGATTCGGTCTC-3′. Primer of BmPRP1: 5′-GGTGCTGTGATGGCGGAGTTC-3′ and 5′-GCATGGCAGTGATGGAGAGGATC-3′. The qPCR reactive program was subjected to 39 cycles at 95 °C for 10 s, 60 °C for 10 s, 72 °C for 30 s, then 95 °C for 10 s, and 65–95 °C for 5 s. The experiment was repeated three times. The silkworm GAPDH (glyceraldehyde-3-phosphate dehydrogenase) gene was taken as the reference gene. The qPCR data were analyzed by using the 2^−ΔΔCt^ method. The means and DMRT (Duncan multiple range test) were evaluated by employing SPSS software (IBM, Armonk, NY, USA).

### 4.6. Homology Modeling and Molecular Docking

The target sequence of BmArgRS and BmLamin-C were acquired from Uniprot, with the Uniprot ID H9JHD3 and H9J2B5. Template crystal structures of RARS and LAMC were identified through BLAST and downloaded from RCSB Protein Data Bank (PDB ID were 4R3Z1 and 6GHD2). Homology modeling and docking were conducted in MOE v2014.09014 (Chemical Computing Group, Montreal, Canada).

### 4.7. Circular Dichroism Spectrum

Circular dichroism (CD) experiments were conducted by Chirascan Plus V100 (Leatherhead, Surrey, United Kingdom) provided by Applied Photophysics Ltd. Analysis processing in 1.0 nm band width. Measurement range include 190~260 nm (far-UV region scan) and 250–340 nm (near-UV region scan). The test was repeated three times. All raw scanning data were processed in Pro-Data Viewer (Applied Photophysics, Leatherhead, Surrey, United Kingdom) with subtract baseline and smoothing analysis.

## Figures and Tables

**Table 1:** Detailed results of bio-layer interferometry (BLI) assay.

Proteins	DA Con. (μM)	Response (nm)	K_on_ (1/Ms) ^1^	K_dis_ (1/s) ^2^	K_D_ (M) ^3^
BmArgRS	25	−0.0006	6.68 × 10^2^	3.70 × 10^−2^	5.53 × 10^−5^
	200	0.0093			
	300	0.0192			
BmLamin-C	25	−0.0015	3.61 × 10^2^	3.12 × 10^−2^	8.64 × 10^−5^
	200	0.008			
	300	0.0148			
BmPRP1	15.6	0.0107	/	/	/
	62.5	−0.0047			
	125	−0.0119			
	500	−0.0422			

^1^ K_on_: association rate constant; ^2^ K_dis_: dissociation rate constant; ^3^ K_D_: affinity constant.
